# Computer-assisted image analysis of agonist-mediated microvascular constriction response in mouse cremaster muscle

**DOI:** 10.1371/journal.pone.0277851

**Published:** 2022-11-17

**Authors:** Ricardo Corro-Hernández, Oscar Aguila-Torres, Amelia Rios, Bruno Escalante, Jesús Santana-Solano

**Affiliations:** Centro de Investigación y de Estudios Avanzados del IPN, Unidad Monterrey, Apodaca, Nuevo León, México; Gifu University School of Medicine Graduate School of Medicine: Gifu Daigaku Igakubu Daigakuin Igakukei Kenkyuka, JAPAN

## Abstract

In this work, we implemented an automated method using a correlation coefficient to select a time interval with a minimum movement or rest interval, together with analysis of variance for measurement of blood vessel diameter in the cremaster muscle. Video images binarization using analysis of variance resulted in an enhanced and a clearly defined vessel wall. Histamine (1 mM) induced a marked reduction in vascular diameter (vasoconstriction) in the cremaster muscle from mice fed with standard (SD) and high fat diet (HFD). However, the effect of histamine was reduced in HFD mice compared to SD mice. Thus, the change in vascular diameter was 87.14% ± 7.44% and 52.63% ± 16.27% in SD and HFD mice, respectively. In conclusion, determination of a rest interval with minimal movement and the use of analysis of variance resulted useful to evaluate vascular diameter in small arteries. We suggest this method to streamline experiments facilitating cardiovascular research.

## Introduction

The microvasculature is a dynamic cellular system, composed of blood vessels ranging in diameter from 5 to 100 μm, that include small arteries and veins, arterioles, venules, and capillaries [1, 2]. Microvascular function depends on the function and structure of the vessel wall. Arterioles are assembled as one to several layers of vascular smooth muscle with an overlaying layer of endothelial cells while the capillary is formed by a single layer of these cells. Regulation of the microvascular function is mostly associated with the endothelial cells by interacting with a variety of endothelial-dependent inflammatory mediators as histamine, serotonin, and bradykinin [3]. This network of microvessels transports and exchanges oxygen and nutrients between blood and tissue, removing CO_2_ and metabolic products and participates in tissue defense and cell repair [3–5]. Alterations in the function of microvasculature including capillary flow velocity and permeability have been associated with several pathological conditions as hypertension [4, 6], and type 1 diabetes [4, 7]. Impaired coronary microcirculation in human has also been associated with obesity [4, 8].

Regulation of blood flow is achieved through changes in the vascular resistance, the force exerted by blood vessels on circulating blood to generate blood pressure [9]. In accord with Poiseuille’s law, very small decrease in vessel radius dramatically reduces flow, demonstrated by the directly proportional relationship between flow and radius to the fourth power, and the inversely proportional relationship between the flow to vessel resistance [10, 11]. The active regulation of vascular resistance is regulated by the vascular tone that in turn depends on blood pressure as well as the balance between vasoconstrictor and vasodilator signals acting on the vessel wall [9, 12]. Arterioles play an important role in controlling peripheral resistance and tissue perfusion. An early report demonstrated the substantial participation of precapillary arterioles with a diameter of 10 to 30 μm in the regulation of the capillary circulation [13]. Therefore, a robust analysis of microvascular regulation requires quantification of changes in vessel diameter and blood flow dynamic conditions [14]. A variety of methods have been used for the measurement of arterial diameter. Manual tracking can be used particularly in isolated blood vessel preparations and requires trained personnel to continuously monitor vascular edges and make manual adjustments. Automated tracking of changes in diameter are based in video images of a transilluminated blood vessels using several thresholding methods to analyze the image. These methods are appropriate for isolated vessels with a clean outer edge [15]. Indeed, *ex vivo* measurements of arterial diameter as indicator of changes in vascular tone have been performed using a combination of video camera and microscope. Pressure myography is one of the most useful tools to measure diameter in small arteries and is widely used in cardiovascular research [16, 17]. The measurement of small arteries diameter in living systems implies several difficulties as the arterioles embedded within a scattering medium or motion of vessels resulting of blood flow and/or respiration. Thus, intravital microscopy and a video caliper with a spatial resolution of ≤ 2 μm have been used to determine in vivo arterioles lumen diameter [18]. An important contribution for determination of vessel diameter and analysis of endothelium-dependent Ca^2+^ signaling in vivo was the use of transgenic mice expressing GCaMP2, a GFP-based Ca^2+^ sensor in vascular endothelium [19, 20]. A similar approach using transgenic mice that express genetically encoded Ca^2+^ biosensors was used to evaluate Ca^2+^ signaling in smooth muscle of skeletal muscle arterioles *in vivo* [21]. Moreover, techniques using intravital microscopy and high-speed digital video system have been developed to study microvascular dynamics. A high-resolution particle image velocimetry (PIV) technique aimed to improve dynamic range, spatial resolution, and measurement accuracy of red blood cells (RBCs) in a rat mesentery arteriole; however, the measurement accuracy of velocity was low [2]. Also, cross-correlation algorithms, a Hough transform method, and an optical flow method have focused on the automatic analysis of microcirculatory blood flow employing microscopic video imaging [14, 22]. However, certain limitations concerning video quality remain to be addressed, particularly in complex vascular structures *in vivo* [23]. Thus, in this work, we used a correlation coefficient to select a time interval with a minimum movement, defined as rest interval, in high-speed videos of mouse cremaster muscle. We also proposed the analysis of variance of gray scales to improve definition of the vessel wall and accurately determine diameter and blood flow velocity in histamine-stimulated cremaster muscle of obese and control mice fed with standard diet.

## Materials and methods

### Cremaster muscle preparation

Male C57BL/6 mice (6–7 week-old) were obtained from the Experimental Animal Care Center from Centro de Investigación y de Estudios Avanzados del Instituto Politécnico Nacional (CINVESTAV-IPN), México. All the procedures were conformed to the National Institutes of Health “Guide for the Care and Use of Laboratory Animals (1996)” and were approved by the Institutional Ethics Review Committee for Animal Experimentation of CINVESTAV-IPN (approval no. 0170–15). All animals had free access to food and water. Mice were divided into two groups: control group fed with standard diet (SD, *n* = 4) and obese group (Research Diets, *n* = 4). Obese animals were fed with a high-fat diet for 8 weeks.

The surgical preparation was performed as previously described [24, 25]. Briefly, mice were anesthetized with ketamine/xylazine (100 mg/10 mg /kg i.p., respectively), additional anesthetic was administered throughout the experiment as needed. A heat lamp was used to maintain mouse temperature. The cremaster muscle was pinned and secured on a PDMS pedestal. Exposed tissue was irrigated continuously with phosphate buffered solution (in mM: 137 NaCl, 4.7 KCl, 1.2 MgSO_4_ 2 CaCl_2_, 10 Na_2_HPO_4_, 18 KH_2_PO_4_, pH 7.4, 37°C). After 20 min of stabilization, the cremaster muscle was stimulated with increasing concentrations (1 uM,10 uM,100 uM and 1 mM) of histamine, recording started 1 min later. Video analysis was performed using 1 mM of histamine that produced the maximal contraction.

Diameter of vessels were measured in microns (μm). To calculate the changes in vascular diameter, data were first normalized considering the basal diameter (μm) as 100%, then reduction of diameter as % was calculated. At the end of the experiment mouse was euthanized by cervical dislocation.

### Statistical analysis

All data are expressed as mean ± standard error of the mean (SEM). The one-way analysis of variance (ANOVA) and Tukey test were used for multiple comparisons. Data were evaluated using GraphPad (GraphPad software, San Diego, CA, USA).

### Intravital microscopy and high-speed video recording

Intravital imaging was performed on an optical microscope (OLYMPUS BX51), equipped with a 100 W halogen lamp, 1.1 N.A. Abbe condenser, and a long working distance ×50 objective (LMPLFLN, infinity-corrected optical system, working distance = 10.6 mm, NA = 0.5, Olympus, Center Valley, PA, USA). With this setup, the resolving power and depth of focus are 0.67 μm and 2.5 μm, respectively. A 335–610 nm bandpass filter (FGB37M, Thorlabs, Newton, NJ, USA) was used to enhance image contrast. After the muscle was exposed, stabilization of 20–30 min was allowed before measurements.

Video frames were recorded with a high-speed video camera (640 × 640 pixels, 2000 frames/s, shutter speed = 1/2000 s, Fastcam 60K-M1, Photron, San Diego, CA, USA). The spatial resolution of the images was 0.34 μm/pixel. The same arterioles chosen at baseline were followed throughout the experiment. Only arterioles in the range of 20 to 30 μm in diameter were used.

### Video analysis

**Fig 1:**
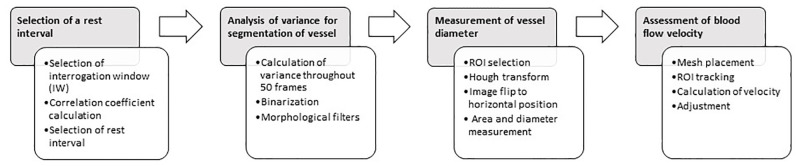
Schematic representation of the algorithm.

The videos were analyzed using a custom-made algorithm implemented in MATLAB (MathWorks, Natick, MA). Fig 1 illustrates the different steps used to measure the diameter and blood flow of arterioles in a mice cremaster muscle preparation.

### Algorithm description

#### A. Selection of a rest interval

1. An interrogation window (IW) with X pixels wide and Y pixels high, for every frame outside the most prominent vessels was selected.

2. The correlation coefficient between frames was calculated:

$$r_{k}=\frac{\sum_{x}\sum_{y}\left(f\left(x,y\right)-\;\bar{f}\right)\left(g\left(x,y\right)-\;\bar{g}\right)}{\sqrt{\left(\sum_{x}\sum_{y}{\left(f\left(x,y\right)-\;\bar{f}\right)}^{2}\right)\left(\sum_{x}\sum_{y}{\left(g\left(x,y\right)-\;\bar{g}\right)}^{2}\right)}}$$

2.1 Image frame k was transformed to a numerical matrix *(x*,*y)* containing the grayscale values from IW. The average values $\bar{f}$ for *f(x*,*y)* and $\bar{g}$ for *g(x*,*y)* were calculated.

2.2 To identify the rest interval (50 frames), the maximum correlation coefficients were determined.

#### B. Analysis of variance of grayscale values for segmentation of the most prominent vessels

1. The variance of the grayscale values of each pixel throughout the 50 frames of the rest interval was calculated:

$$\sigma^{2}=\frac{1}{N-1}\overset{N}{\underset{i=1}{\sum }}{\left|s_{i}\;-\;\bar{s}\right|}^{2},$$

where *N* is the number of frames, *s*_*i*_ is the grayscale value of frame *i*, and $\bar{s}$ is the mean grayscale of the *N* frames. This was repeated for every pixel in the rest interval. At the end, an image with the variance of grayscale values in this interval was obtained.

2. The image obtained with the variance of each pixel was binarized by selecting a gray level threshold.

3. Additional image processing using morphological filters was performed to eliminate noise outside blood vessels.

#### C. Measurement of vessel diameter

1. A region of interest (ROI) in the binarized image of the rest interval frame was manually selected.

2. The Hough transform was used for detecting straight lines and calculate the orientation angle and rotate the image to display the ROI in horizontal position.

3. The white pixels were counted to calculate the area of the rotated image. Vessel diameter was determined using this area and the length of the analyzed segment.

#### D. Assessment of blood flow

1. A mesh of 15 x 5 IWs of 20 x 20 pixels (46.24 μm^2^), that is close to the physical size of an RBC was generated in the previously selected ROI (in horizontal position) of the vessel. These IWs automatically started at the center of vessel continuing towards the vessel wall. To obtain a high density of velocity vectors, IWs were partially overlapped depending on the diameter of arteriole.

2. Once IWs were located, they were tracked in the subsequent images using the normalized 2-D cross-correlation:

$$\gamma \left(u,v\right)=\frac{\sum_{x,y}\left[f\left(x,y\right)-\;{\bar{f}}_{u,v}\right]\left[\text{IW}\left(x+u,y+v\right)-\bar{\text{IW}}\right]}{{\left\{\sum_{x,y}{\left[\left[f\left(x,y\right)-{\bar{f}}_{u,v}\right]\right]}^{2}\sum_{x,y}{\left[\text{IW}\left(x+u,y+v\right)-\bar{\text{IW}}\right]}^{2}\right\}}^{0.5}},$$

where *f*(*x*,*y*) indicates the intensity level of grayscale at coordinates *x*, *y* of the searching area, IW is the interrogation window or template, and (*u*, *v*) are the shift indexes. ${\bar{f}}_{u,v}$ and $\bar{\text{IW}}$ are average intensities of the windows.

3. The velocity was calculated using the displacement from the initial position of the IW and the time between frames (determined by the camera frame rate).

Spurious velocity vectors were eliminated using Tukey´s fences to determine outliers below the first quartile and above the third quartile, since the normalized 2-D cross-correlation particle image velocimetry (PIV) is prone to generate spurious velocity vectors due to noise on images or/and intrinsic limitations of the algorithm.

4. To calculate blood flow, the average velocity of RBCs in the ROI was determined. First, the time-average velocity profile of the 5 columns in the IW were calculated. Then, a spatial average of the 5 velocity profiles was performed to obtain a single velocity profile.

The average velocity profile was fit to the following function:

$$V=\;V_{max}*\left(1\;-\;{\left(\frac{r}{R}\right)}^{k}\right),$$

where *V*_*max*_ is the maximum speed value, *r* is the radial coordinate from the arteriole center, R indicates average radius of the arteriole in the ROI, and *k* is the bluntness index. The best fit for *k* was evaluated by using the least squares method.

## Results

### Image processing

**Fig 2:**
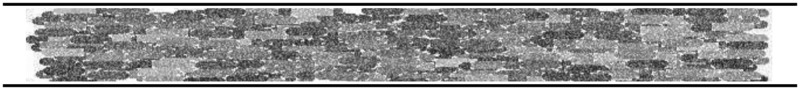
Simulated blood flow. Representative image of computer-generated simulation of the blood flow.

The algorithm used in this work was validated by videos generated by computer simulating the flow of RBCs and the known displacement between frames. A representative image of a frame from the blood flow simulation shows the complex layout of blood vessels (Fig 2).

**Fig 3:**
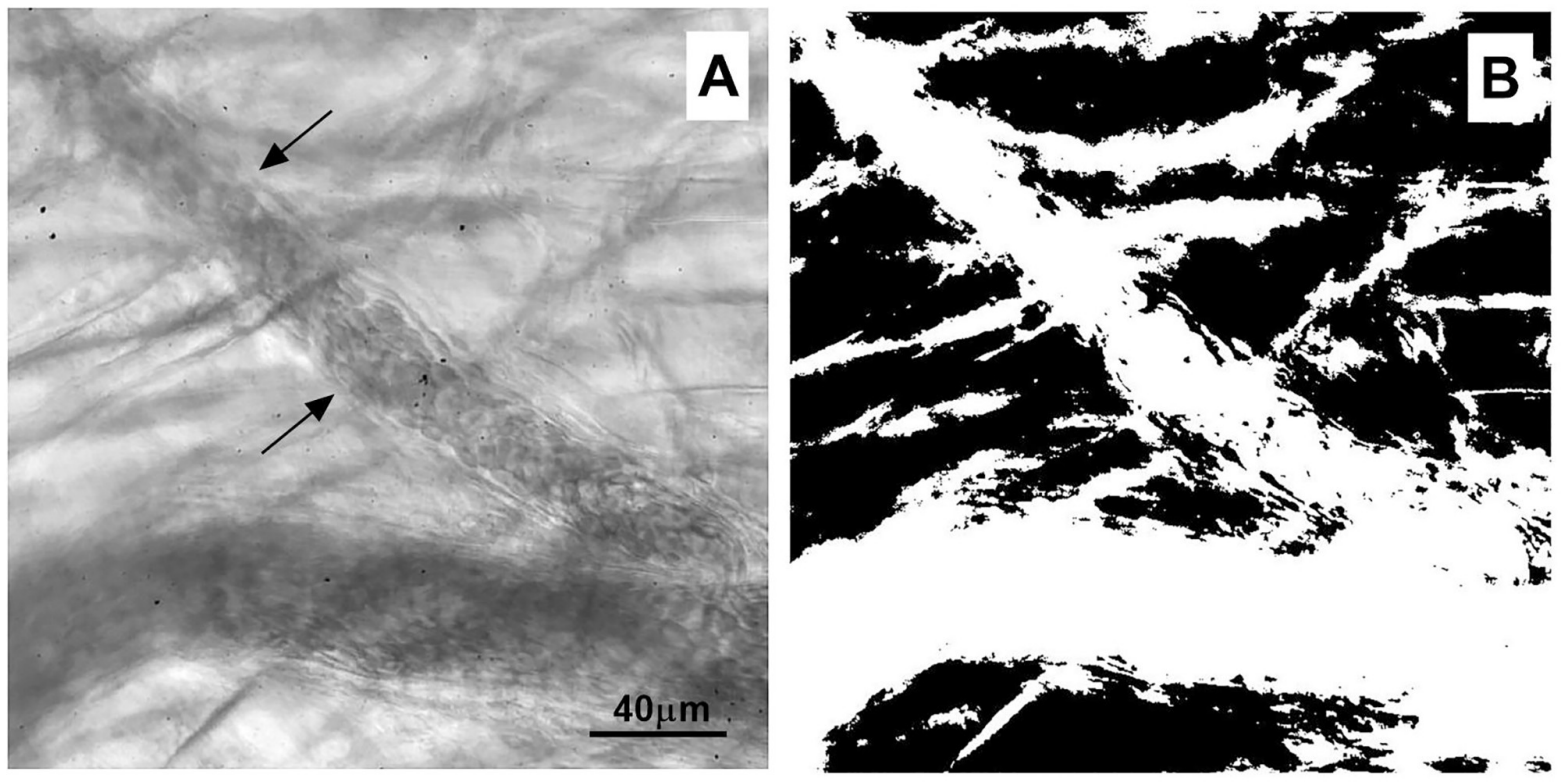
Thresholding. (A) ROI of an arteriole in mouse cremaster muscle. This image depicts the poor definition of the blood vessel border (arrows). (B) Segmentation of image with a global thresholding sharpened the vessel body, however the vessel wall remained poorly defined.

In the cremaster muscle, under basal conditions, the border of blood vessels can be observed, however likeness of grayscale values complicates definition of the wall (Fig 3A). A global thresholding segmentation on the image helped to sharpen the body vessel, however the vessel wall was poorly defined (Fig 3B). Thus, the lack of good contrast close to the vessel wall makes difficult to precisely calculate the blood vessel diameter. This effect is particularly relevant in analysis of *in vivo* preparations mainly due to the blurriness associated with movement of the tissue.

**Fig 4:**
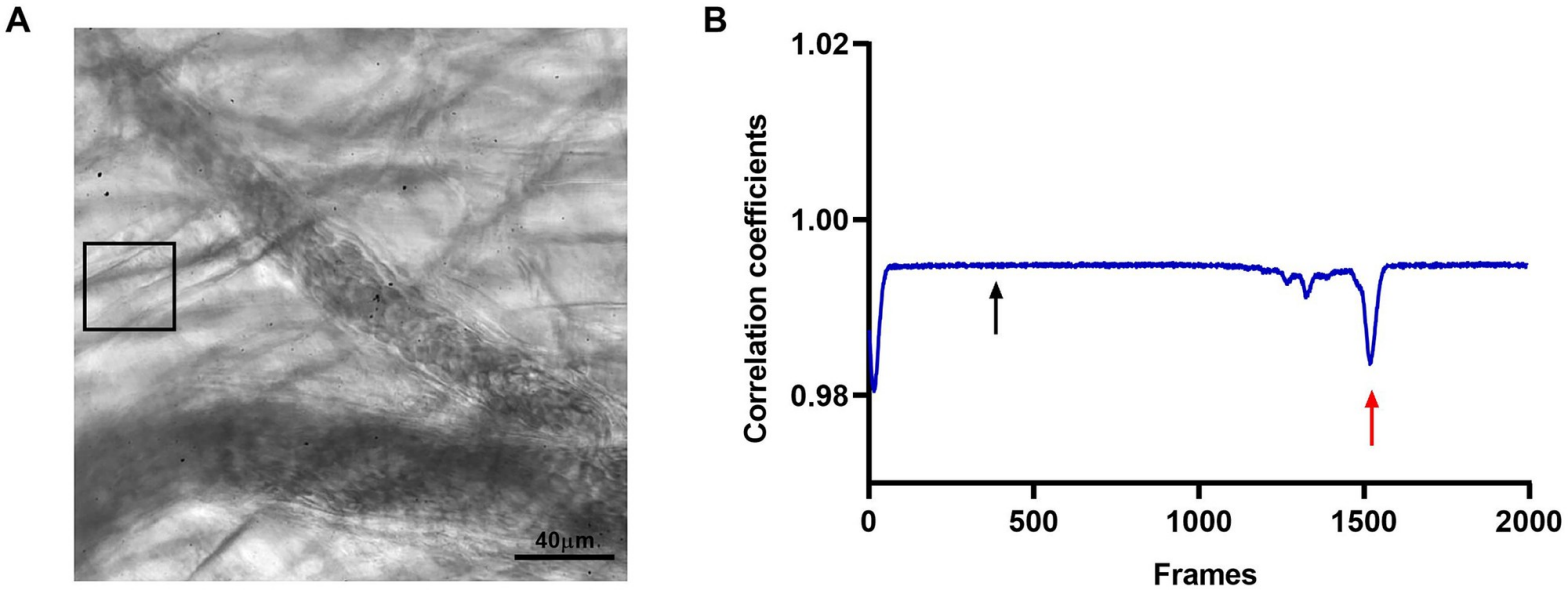
Rest interval and correlation coefficients. (A) A rest interval (black square) meaning the area with the least movement, was selected in the ROI. (B) Graph showing the calculated correlation coefficients. The black arrow shows the highest and constant values of correlation coefficients. The red arrow shows the peak generated by muscle contraction.

To overcome this effect, we selected within the video, a time interval with a minimum movement (rest interval). An interrogation window was selected outside the largest blood vessel (Fig 4A). The calculated correlation coefficients (Fig 4B) with the highest values correspond to absence of movement where the function has constant values (black arrow). In addition, the lowest values of the correlation coefficient indicate the time span of muscle contraction (red arrow).

**Fig 5:**
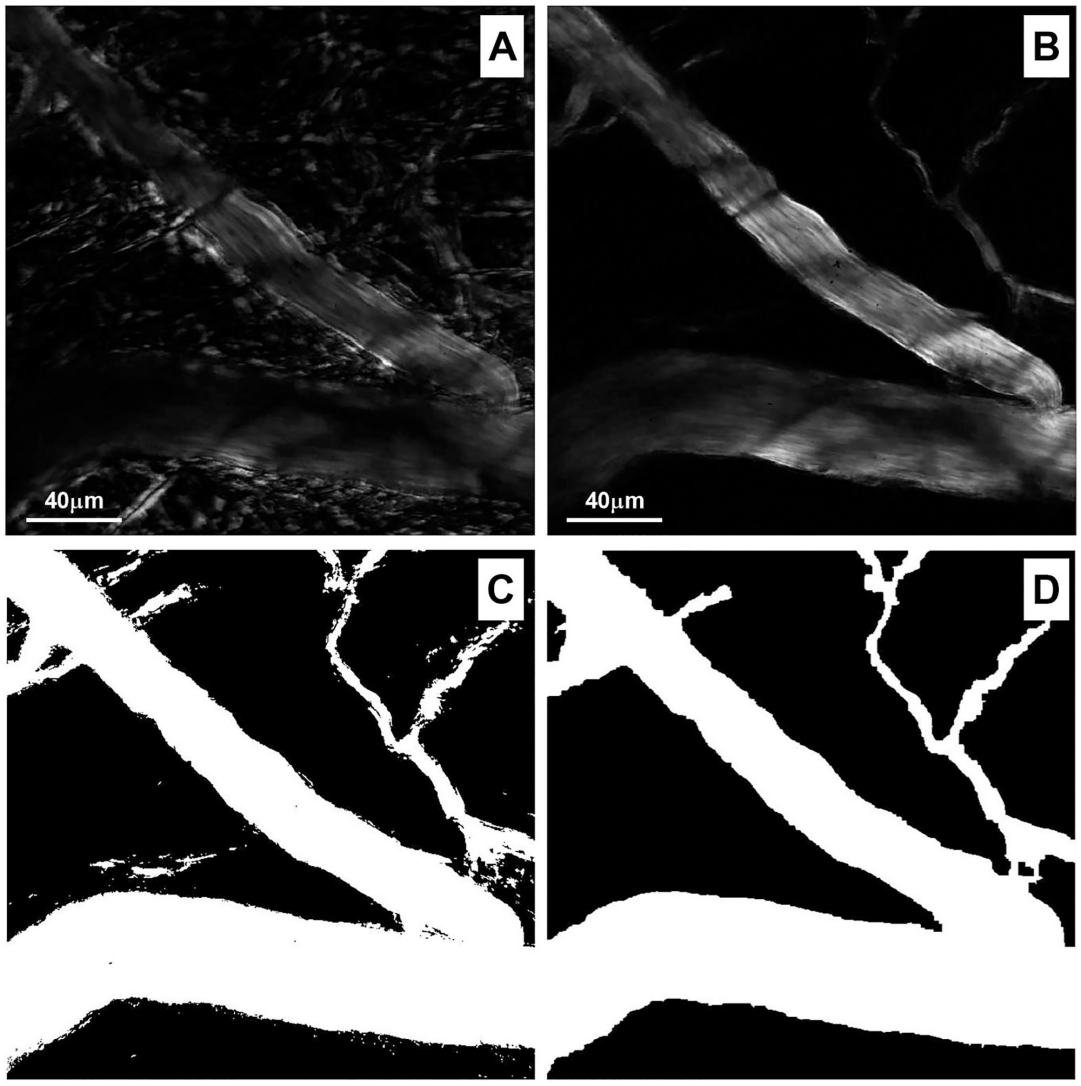
Enhancement of image. (A) The rest interval and analysis of variance improved the outline of the blood vessel. (B) Image of arteriole without rest interval. (C) Binarization of the image (D) A morphological filter applied to the binarized image.

Subsequently, blood vessel image was enhanced with analysis of variance of the gray scale values. Fig 5A shows the arteriole chosen ROI without rest interval. The same image using the rest interval is shown in Fig 5B. Binarization of this image with analysis of variance is shown in Fig 5C, then a morphological filter was applied (Fig 5D). These steps enhanced the image and clearly defined the vessel wall.

### Measurement of blood vessel diameter

**Fig 6:**
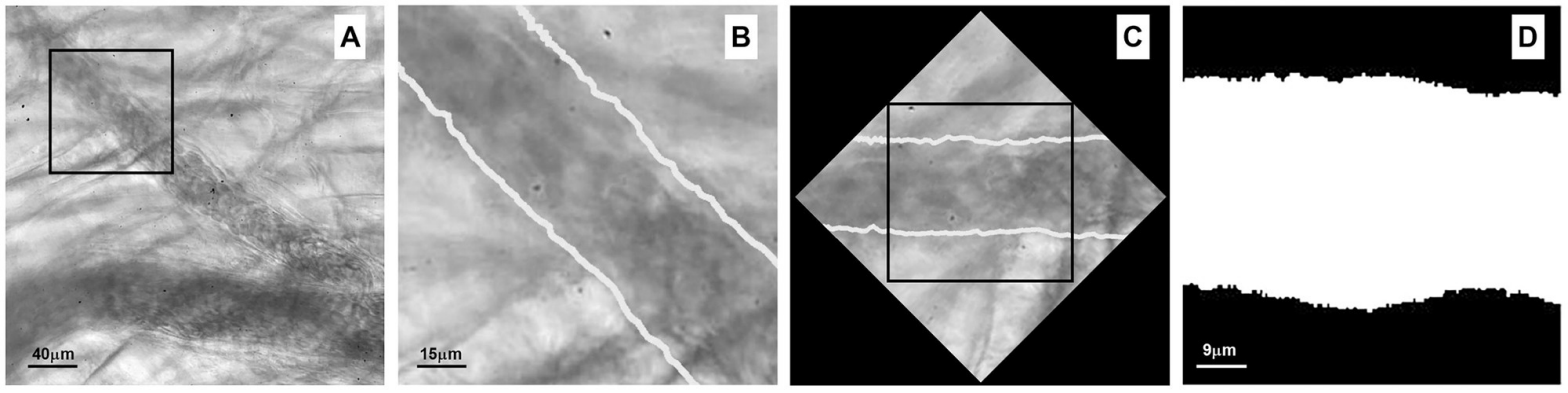
Measurement of the arteriole diameter. (A) To measure the diameter of arteriole, a sub-image of 100 x 100 pixels was generated (black square). (B) In this new image, the vessel wall was highlighted in white. (C) The Hugh transform was applied, and the image rotated to horizontal position. (D) The arteriole diameter was calculated in the binarized image.

After segmentation of blood vessels, the diameter of arteriole was calculated in a sub-image of 100 x 100 pixels (1156 μm^2^, Fig 6A). The irregular pattern of the vessel wall was highlighted in white (Fig 6B). By applying the Hough transform, we calculated the angle of orientation of the blood vessel that allowed us to rotate the image to a horizontal position (Fig 6C). The angle with respect to the horizontal was of 140°. The arteriole average diameter measured in the binarized image was 27.5 μm (Fig 6D).

### Blood flow velocity detection

**Fig 7:**
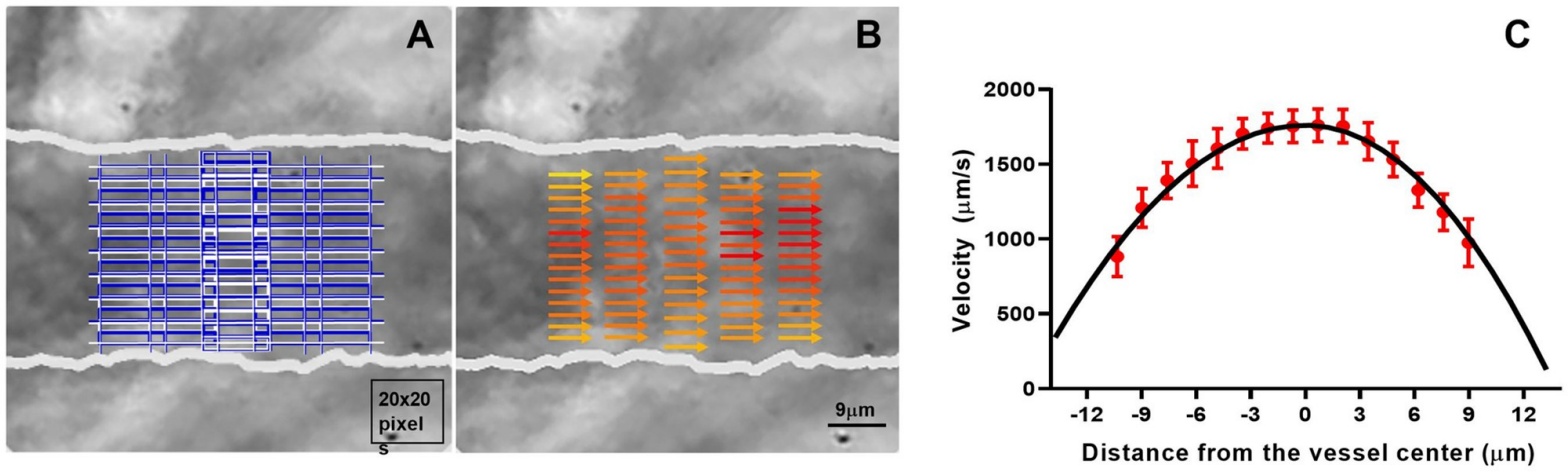
Measurement of blood flow velocity. (A) Automated generation of a mesh in the lumen of the blood vessel sub-image for measurement of blood flow. (B) Velocity vectors indicates the direction of blood flow. Color ranges from light yellow at the minimum velocity to deep red at maximum velocity detected. (C) Measured speeds (red dots) were fitted with the parameter *k =* 2 (black curve).

To determine blood flow velocity, a mesh was automatically placed in the blood vessel lumen. This mesh consisted of 5 columns of interrogation windows partially overlapped and bounded by the vessel walls (Fig 7A). The velocity vectors (Fig 7B) showed some asymmetric velocity profiles that might be due to the variability of diameter along the blood vessel. Velocity profiles also showed a nearly parabolic shape (Fig 7C). The distribution of these velocity profiles along the segment of blood vessel analyzed was inversely proportional to the diameter of the arteriole.

The mean blood flow velocity of RBCs in the third and fifth columns were 1312.8 μm/s and 1787.6 μm/s, respectively, corresponding to vessel diameters of 30.3 and 25.2 μm, respectively.

The mean blood flow velocity in the ROI was obtained averaging velocity profiles from 5 columns of velocity vectors. This mean blood flow velocity was fitted using eq. 4 and the resultant parameter *k* was 2.

### Effect of histamine on cremaster muscle arterioles

We evaluated the effect of histamine in the microcirculation of mice cremaster muscle. Histamine can influence tissue blood flow eliciting contraction by activation of H1-receptors [18, 26]. Hence, we measured diameter and blood flow velocity in arterioles from the cremaster muscle from SD and obese mice using our automatized method of binarization.

**Fig 8:**
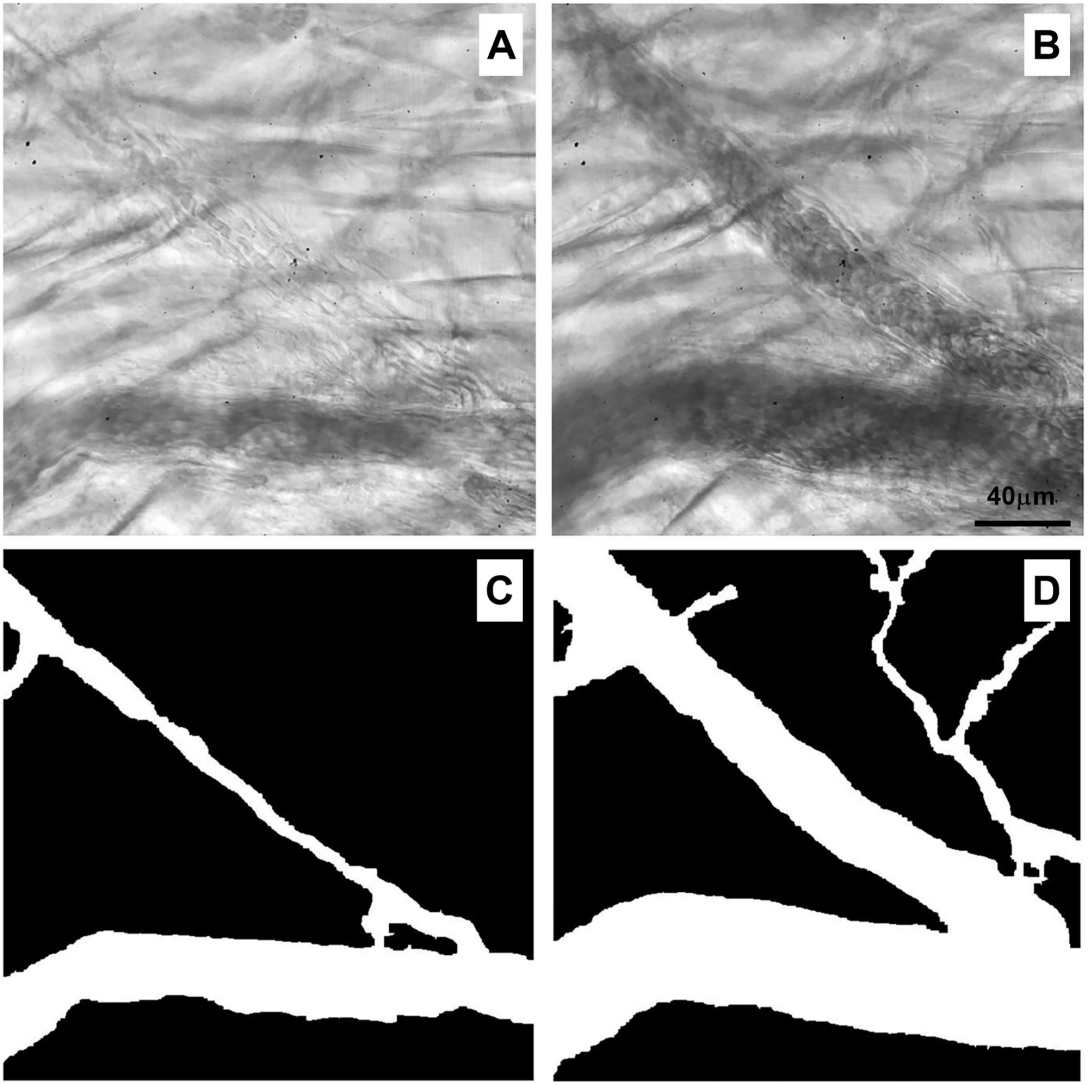
Effect of histamine on microcirculation of mice cremaster muscle. (A) Contracted arteriole elicited by stimulation with histamine (1 mM). (B) Arteriole in basal condition. (C) Processed image of arteriole stimulated with histamine. (D) Processed image of arteriole in basal condition.

Stimulation with histamine (1 mM) elicited a marked contraction of arterioles (Fig 8A). Under this condition, presence of the vessel wall and RBCs flow was unclear compared with basal condition without histamine (Fig 8B). Processed and enhanced images allowed a precise measurement of the reduced diameter in blood vessel stimulated with histamine (3.46 ± 4.01 μm, Fig 8C) compared to vessel in basal condition (26.28 ± 0.77 μm, Fig 8D). Blood flow velocity was decreased from 1423.3 ± 844.33 μm/s to 570.77 ± 717.47 μm/s after histamine administration.

**Fig 9:**
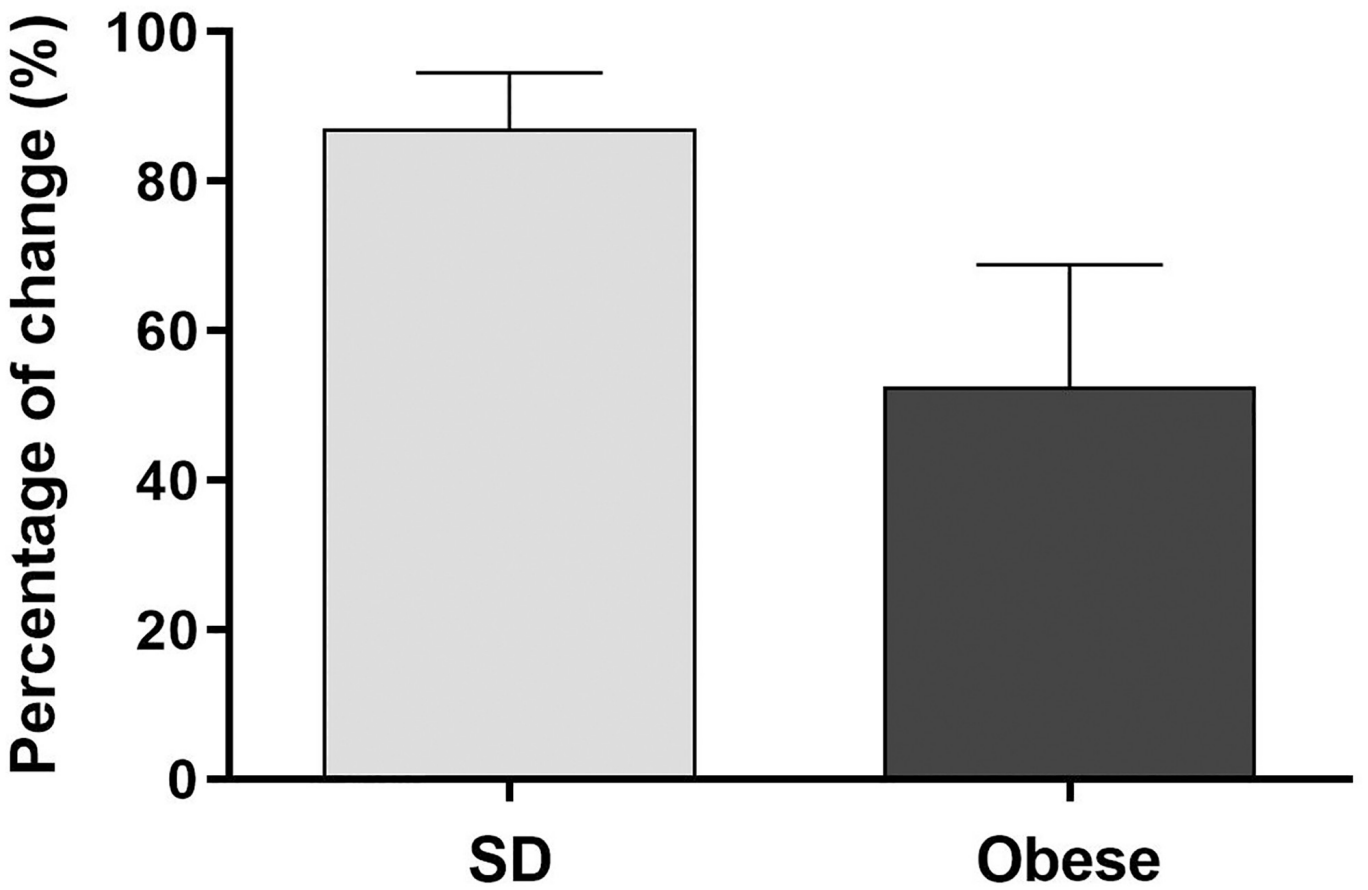
Effect of histamine in SD mice compared to obese mice. The graph illustrates the percentage of change (%) in diameter of SD mice compared to obese mice after stimulation with histamine. *n* = 4 mice per group. Data represent the mean ± SEM.

Furthermore, our automated image analysis allowed us to compare vascular response to histamine in arterioles from SD and obese mice (Fig 9). Histamine promoted vasoconstriction in both groups, however the change in the response was different. Thus, the change in the vasoconstriction response dependent of histamine was reduced in arterioles from obese mice (52.63% ± 16.27%) compared to arterioles from SD mice (87.14% ± 7.44%).

**Fig 10:**
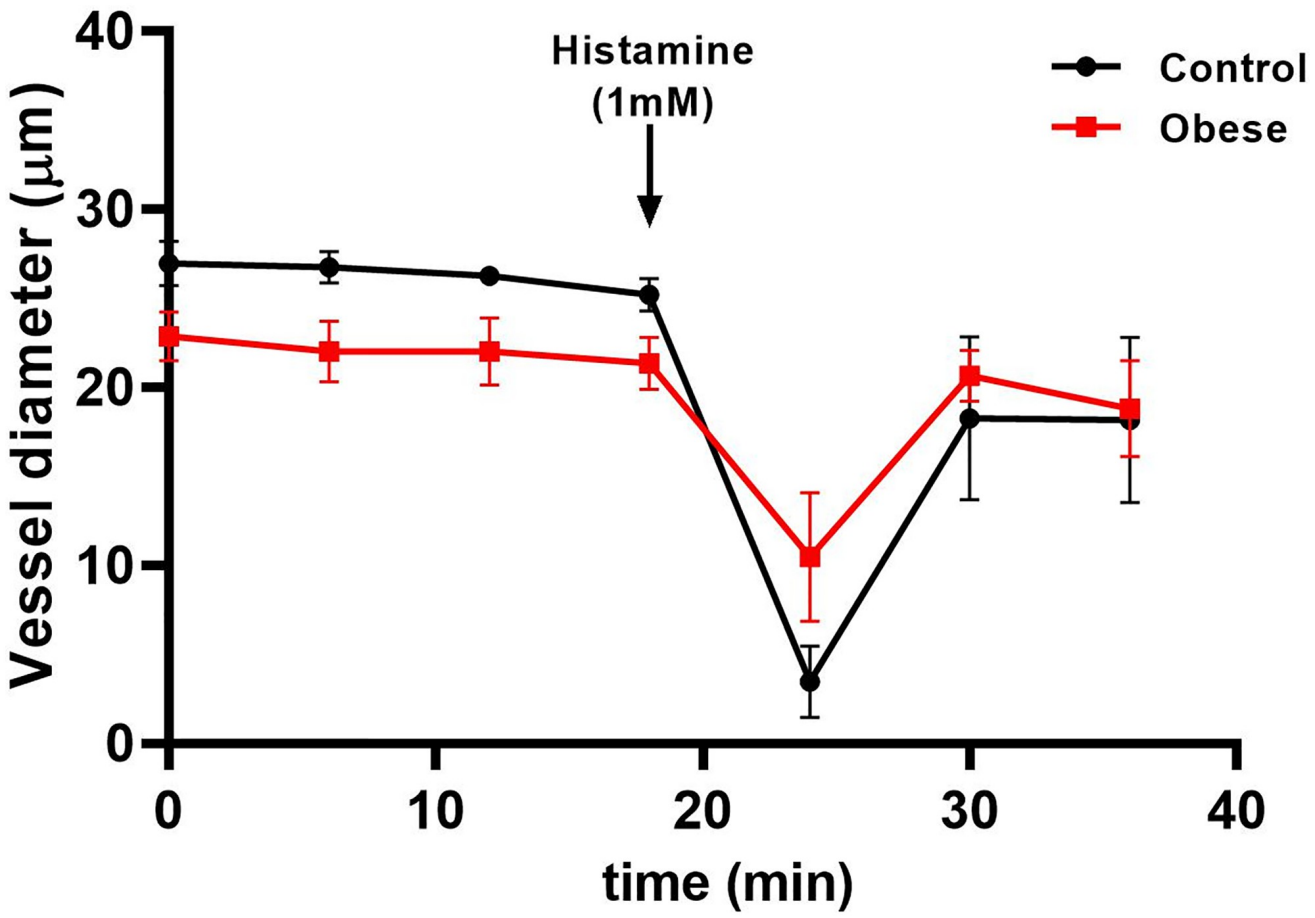
Time- course of histamine effect on vessel diameter. Microvessels diameter of SD and HFD mice cremaster muscle was evaluated during 35 min. A vasoconstrictor effect was observed after histamine (1 mM) stimulation (arrow) followed by vasodilation after histamine suspension. Data represent the mean ± standard error of four different experiments. *p ≤ 0.05 vs control.

In addition, a time-course evaluation showed reduction in the vessel diameter after stimulation with histamine (1 mM) followed by time-dependent vasodilation of the vessel diameter (Fig 10).

## Discussion

The goal of this study was to develop an improved image processing system for accurate measurement of microvessels diameter and blood flow velocity in mice cremaster muscle. This improved system would permit to evaluate the functional response of arterioles from control and obese mice to physiological agonist histamine. Here we report a new processing algorithm to track changes in diameter of arterioles and analyze blood flow velocity. The achievement presented here is based on determination of the variance of greyscale values and the selection of frames in a location of tissue without movement that we called rest interval. The quality of video images was improved obtaining images of arterioles with well-defined shapes and borders providing greater accuracy for measurements.

Previous *in vivo* and *in vitro* techniques have been used to study microvascular dynamics including laser Doppler velocimetry [27], high-resolution particle image velocimetry technique (PIV, [2, 28]) and a modified PIV version [16]. Tsukada et al. [29] reported an automated system for measuring blood flow velocity by image correlation using a circular window, sized equal to that of erythrocyte size. Analysis of microcirculatory blood flow in a rat cremaster muscle preparation using a cross-correlation algorithm has also been reported [14]. However, some limitations for these methods are the necessity to use fluorescent markers, the error caused by movements of the specimen or the requirement of good video images showing venules and arterioles with well-defined shapes and borders [14, 30]. Simulations have been also used to analyze microcirculation. Simulated microscopic video image sequences have been evaluated with different methods including cross correlation [14], Hough transform [22], optical flow [22], Adaptive Window based Cross-Correlation (AWCC) scheme [22, 23], and smoothed dissipative particle dynamics (SDPD, [1]). These methods may improve accuracy of measurements but can lack of correlation with in vivo complex vascular structures.

Thus, quantitative analysis of in vivo microcirculatory function is relevant to dissect and understand mechanisms involved in physiological and pathophysiological cardiovascular regulation [4, 31].

Since several factors affect blood flow in the microvessel, microcirculation does not necessarily follow the flow velocity profile based on Poiseuille’s law.

The contractile capacities of the vascular tissue, and the fast response to physiological or pathological stimulus that modify the vascular diameter led to the conclusion that vascular regulation may be determined by changes in vessel diameter [32]. Indeed, Jeong et al. demonstrated in vivo that variability of capillary diameter rapidly affected the deformation of red blood cells [33]. Automated tracking of vascular diameter is usually based in video images of a transilluminated blood vessel, and edge transitions are determined against a typically lighter background [15]. However, edge limits can be difficult to determine due to motion of the vessel related to blood flow or respiration of the animal. An automated detection and analysis of vessel diameter could avoid unnecessary work, thus increasing the rate of success with experiments. Previous studies have demonstrated that edges of blood vessels can be clearly defined using transgenic mice [19, 20, 21]. However, the use of a transgenic mice may not be widely available particularly in developing countries. Thus, in the present study we propose the selection of a time interval with minimal movement to automatically compensate for image drift or vessel wall distortion that resulted in vessel images with more clear and defined edges. Although human pattern recognition ability is used in the first frame, the rest of the analysis is fully automated, thus no trained personnel would be required to make manual adjustments to measure the vessel diameter. Furthermore, this method tracks and measures diameters along the full vessel segment of the selected image.

This manuscript presents a methodology to improve the processing of images for accurate measurement of microvessel diameter and blood velocity evaluation. We used intravital microscopy combined with high-speed video recordings (2000 fps) for the analysis of microhemodynamic activities including vasomotion and vasoconstriction in response to histamine, a physiological agonist. The proposed method was validated by our results. Blood vessel diameters were obtained in straight arteriolar segments varying from 25.45 to 27.18 μm under basal conditions. Our data are consistent with previous reports [32]. A possible disadvantage in our method is when vessels constrict to minimum diameter and the image become blurry and noisier. This effect has also been reported by others [34]. However, in our data the maximal reduction in vessel diameter was 80% and our method was able to determine blood vessel edges.

Analysis of hemodynamic conditions under vascular agonist stimulation showed a clear vasoconstriction of arterioles in the mice cremaster muscle treated with histamine. The analysis of blood vessels response to vascular agonists as histamine and the concomitant relationship with blood flow velocity has great implications for understanding cardiovascular physiology [7, 8, 10, 35]. Alterations in microvascular blood flow are frequently observed in patients with severe heart failure [36]. Impaired tissue perfusion caused by abnormality of the microvascular system has also been observed as long-term complication of hypertension and diabetes [4]. Histamine is a modulator of blood flow. Several reports have shown that histamine promotes arteriolar dilation while others have described vasoconstrictor effect of histamine. Thus, the effect of histamine on vessel diameter and blood flow may be dependent on the concentration [18, 37]. Our results showed that stimulation with histamine (1 mM) resulted in arteriolar constriction associated with reduced vessel diameter and decreased blood flow. This result is supported by Payne et al. [18] that reported histamine vasoconstrictor effect at concentration > 1 μM. In basal conditions, image of blood vessel is well defined (Fig 7A). However, after stimulation with histamine, image of the constricted arteriole is not well delimited, particularly the border is almost impossible to find (Fig 7B). Thus, use of the computer-based method, based on determination of the variance of greyscale values and the selection of a rest interval, resulted in a clearly defined image that allowed evaluation of the border wall *in vivo*. A previous work reported a software for measurement of the diameter of isolated retinal arterioles, with no need for manual definition of the initial window or highlighted of the walls for vessel analysis [38]. More recently, a study using the neural network U-Net architecture for vessel segmentation achieved discrimination of artery/vein and the automated measurement of isolated mouse small mesenteric arteries [39]. Although these last studies were achieved in isolated vessels, the manual selection of vessel as well as the rest interval are limitations of our method that still need to be elucidated.

## Conclusion

In conclusion, we propose an automatized method with higher resolution to evaluate in vivo hemodynamics from microcirculation video recordings. This approach was capable of segmenting blood vessels of mice cremaster muscle over a time interval where the muscle is at rest. It was based on the analysis of variance of gray levels generated by blood flow. The presented method allowed us to perform an accurate segmentation of vessels even under very low contrast conditions particularly observed in the smallest blood vessels. Once the inner vessel wall was found, a region of interest was selected for measurement of both vessel diameter and blood velocity in that region. The blood velocity profile was locally asymmetric along the vessels, and the maximum velocity was inversely correlated with the local diameter of the vessel. This work further analyzed the response to histamine of blood vessels of cremaster muscle, demonstrating that vasoconstriction response, expressed as change of the response, was reduced in obese mice compared to control animals.

Future work will focus on extending the analysis of blood velocity profiles using this segmentation approach to include bifurcations of small arterioles produced after the contraction induced by the physiological agonist histamine. The method proposed here would be particularly useful to address microvascular pathological conditions related with severe heart failure or hypertension.

## Supporting information

S1 Data Velocity profile analysis. (PZF)

S2 Data Response to histamine. (PZF)

S3 Data Velocity profile. (PZF)

S1 Video Arteriole of mice cremaster muscle in vivo. (AVI)

S2 Video Histamine-stimulated arteriole of mice cremaster muscle in vivo. (AVI)

S3 Video Highlighting of the blood vessel border. (AVI)

S4 Video Highlighting of the blood vessel border after histamine stimulation. (AVI)
